# Undifferentiated Sarcomatoid Carcinoma of the Pancreas: From Histology and Molecular Pathology to Precision Oncology

**DOI:** 10.3390/ijms23031283

**Published:** 2022-01-24

**Authors:** Anastasios Gkountakos, Michele Simbolo, Elena Bariani, Aldo Scarpa, Claudio Luchini

**Affiliations:** 1 ARC-NET Applied Research on Cancer Center, University of Verona, 37134 Verona, Italy; anastasios.gkountakos@univr.it (A.G.); aldo.scarpa@univr.it (A.S.); 2 Department of Diagnostics and Public Health, Section of Pathology, University of Verona, 37134 Verona, Italy; michele.simbolo@univr.it (M.S.); elena.bariani@studenti.univr.it (E.B.)

**Keywords:** sarcomatoid, mesenchymal, EMT, PDAC, pancreatic ductal adenocarcinoma

## Abstract

Undifferentiated sarcomatoid carcinoma of the pancreas (SCP) is a rare and aggressive subtype of pancreatic cancer. Histologically, SCP is a poorly differentiated tumor characterized by the lack of glandular differentiation and the presence of mesenchymal-like, spindle-shaped tumor cells. Due to its rarity, only sporadic cases have been reported, while its molecular characterization has not been sufficiently described. Surgical resection with curative intent is the gold-standard of SCP management, but this strategy is possible only in a small proportion of cases due to SCP early metastasization. Although SCP is generally associated with a poor prognosis, some clinical cases amenable to surgical resection and followed by adjuvant chemotherapy have demonstrated a remarkably long survival. Preliminary molecular insights on the SCP molecular landscape have demonstrated the recurrent presence of *KRAS* and *TP53* mutations, highlighting genetic similarities with conventional pancreatic ductal adenocarcinoma (PDAC). Although the use of immunotherapy in PDAC remains an unmet challenge, recent insights indicated a potentially significant role of the PD-L1/Notch3 axis in SCP, opening new horizons for immunotherapy in this cancer subtype. In this review, we described the most important clinic-pathologic features of SCP, with a specific focus on their molecular landscape and the potential targets for precision oncology.

## 1. Introduction

**Figure 1:**
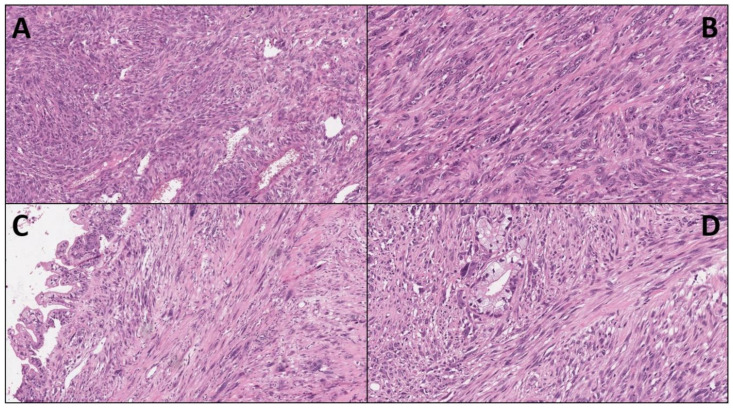
Four paradigmatic images of undifferentiated sarcomatoid carcinoma of the pancreas are presented (Hematoxylin-eosin): (**A**) typical hyper-cellular appearance (original magnification × 10); (**B**) the spindle-shape and the high-grade of atypia of sarcomatoid cells (×20); (**C**,**D**) these images are Scheme 20.

Pancreatic cancer (PC) is one of the deadliest cancer types, with a constantly increasing incidence [1,2]. Pancreatic ductal adenocarcinoma (PDAC) is the most-common type of PC, accounting for more than 90% of cases and showing a very low survival rate (<10% 5-year overall survival) [3]. This very poor prognosis is mainly due to the difficulties for early-stage detection of tumors as well as the very-aggressive cancer biology [4]. Despite the constant and intensive efforts to develop new therapeutic approaches, current treatment options have struggled to significantly prolong patients’ survival. The standard of care for patients with PC remains surgery and chemotherapy/radiotherapy. However, most patients due to late diagnosis are not eligible for surgery, while they also rapidly develop resistance to chemotherapeutic regimens [5]. Interestingly, immune-checkpoint inhibitors for cancers with microsatellite instability, as well as targeted therapies such as those based on PARP inhibitors for *BRCA*-mutated tumors, are gradually entering into clinical practice, but these opportunities regard only a small proportion of PDAC patients [6,7,8,9,10]. Beyond PDAC, several other morphologically diverse subtypes of PC have been identified. Among these, sarcomatoid carcinoma of the pancreas (SCP) is an extremely rare but very aggressive subtype with a poor prognosis. According to the 2019-WHO classification, SCP represents a subtype of undifferentiated PDAC and accounts for up to 2–3% of all PDAC and its variants [11,12,13]. Histologically, SCP is predominantly composed of neoplastic spindle-shaped cells with epithelial derivation, showing both epithelial and mesenchymal features (Figure 1) [14,15,16]. Owing to the rarity of the disease, all the available information is originated from individual case reports or small patients’ cohorts. Therefore, the clinic-pathologic features, the molecular landscape, and the therapeutic strategies are poorly characterized. In this review, we aimed at describing the most-important histopathological and clinical features of SCP, with a specific focus on their molecular landscape and the potential targets for precision oncology.

## 2. Undifferentiated Sarcomatoid Carcinoma of the Pancreas: What Do We Know So Far?

Overall, the general knowledge on SCP is limited and can be derived mainly from single case-reports. Therefore, we performed a literature review using PubMed and SCOPUS up to 15 December 2021 with a combination of all these keywords: “pancreatic”, “sarcomatoid”, “cancer” and “adenocarcinoma” but without the specific aim to be strictly systematic. To be considered eligible for this review, the studies must report survival data; moreover, a specific histopathologic diagnosis of SCP was required.

In the main body of this review, we start with presenting the most paradigmatic cases of SCP (summarized in Table 1), in increasing order of survival. Then, we highlight the lessons that can be learned from such cases and their management, also focusing on molecular pathology and clinical implications.

The first case regarded a 39-year-old male, with a rapidly growing pancreatic lesion, initially measured as 2 × 3 cm that increased to 5 × 5 cm in 15 days and then subsequently to 7 × 10 cm within the following 10 days. The patient eventually passed away approximately one month after the first diagnosis. After histological examination, the tumor was identified as an adenosquamous PDAC with the presence of sarcomatoid transformation. A further confirmation of the occurrence of a sarcomatoid component was provided by immunohistochemistry (IHC), where a diffuse vimentin positivity was detected in the area including spindle-shaped neoplastic cells. Interestingly, the high proliferation Ki-67 index (80%) together with the activation of the biological mechanism of epithelial-to-mesenchymal transition (EMT), a crucial biological event in PDAC and in particular in undifferentiated PDAC here demonstrated by the diffuse vimentin expression [15,16], suggested an aggressive biology, likely explaining the rapid worsening of the clinical situation of the patient and eventually his death [17].

A subsequent report described another adenosquamous PDAC with the occurrence of sarcomatoid differentiation, suggesting the presence of a tumor with three different histopathological subtypes: ductal, squamous, and spindle-cells. IHC identified the expression of vimentin and the absence of E-cadherin in the sarcomatoid component. Conversely, the adenosquamous area strongly expressed E-cadherin and lacked vimentin expression, highlighting the divergent differentiation of tumor cell-population. Interestingly, p63 was highly expressed in the squamous part and not (absent-to-very low level) in sarcomatoid cells. The patient developed metastases 5 months after surgery, but there are no data on the long-term follow-up [18].

The case of a 48-year-old male is also of interest; the histopathologic diagnosis of SCP was based on the presence of neoplastic spindle and giant cells with a diffuse vimentin expression and no expression of cytokeratin (CK) 18. Although the patient underwent a radical surgical excision and received adjuvant chemotherapy, he displayed a very poor prognosis, dying 3 months after the operation [19]. Along this line, another case report of SCP confirmed the same IHC profile, with sarcomatoid cells positive for vimentin and negative for epithelial markers. Here, the patient refused surgical treatment and succumbed to the disease within 3 months [20]. Interestingly, a subsequent report of a 63-year-old male patient with SCP suggested that sarcomatoid spindle cells might express both epithelial (CK7 and CK19) and mesenchymal markers (vimentin). The patient underwent surgical resection with curative intent but died after 18 months due to liver failure for multiple hepatic metastases [21].

In another paradigmatic case, a 64-year-old male with a SCP was presented. The tumor was surgically resected, and the histological examination detected the presence of two tumor cell sub-populations, one with an epithelial origin and one with mesenchymal differentiation. The immunohistochemical characterization of the epithelial compartment, mainly represented by undifferentiated cells but also by malignant glands, revealed a strong positivity for CK18 and the lack of expression of mesenchymal markers, such as vimentin; at the same time, the sarcomatoid part was diffusely positive for vimentin. Interestingly, the patient was still alive and free-of-disease 19 months after surgery [22].

Another case that exhibited interesting data regarded a 61-year-old woman, who underwent surgical resection of SCP. The resection was R0. The patient did not receive adjuvant chemotherapy, and she is alive and free of disease 35 months after surgical resection. IHC showed the concomitant positivity of CK and vimentin in tumor cells [23].

It is also of importance reporting the findings of a retrospective review of a mono-institutional case-series, where Blair et al. described the features of more than 7000 pancreatic resections performed in a timeframe of 25 years in their institution. Among them, eight cases (0.11%) were definitively diagnosed as SCP at histology. Six patients underwent R0 resection, and two of them achieved a long-term survival (overall survival > 5 years) [24]. At the same time, two SCP patients who underwent R1 and R2 resection presented early recurrences and experienced a very poor survival (overall survival < 3 months). Although the survival rates of SCP are usually considered worse than PDAC, the authors here suggested that R0 resection together with adjuvant treatment (chemotherapy and/or radiotherapy) might help patients in achieving a longer survival [24].

Another case report is paradigmatic since it investigated TGF-β and IL-11 levels in the plasma of a patient with SCP. The plasma levels of such biomarkers were higher than the normal limits, suggesting the involvement of an EMT-related signaling pathway in SCP. The patient underwent surgical resection. Histologically and immunohistochemically, the diagnosis was of SCP with the concomitant expression of both epithelial and mesenchymal markers. Specifically, tumor cells were positive for CK 18/19 as well as vimentin, supporting the EMT-mediated mesenchymal state. Of note, the patient was still alive and without evidence of disease for more than 3 years after the surgical resection [25].

Another case of SCP was identified by histology coupled with IHC, showing the expression of CK, vimentin, and of two additional markers strictly associated with EMT activation, such as Snail and fibronectin. This patient showed a remarkably long-term survival, being still alive and with no evidence of relapse 10 years after the initial treatment, which was based on radical surgical resection and a 6-month scheme of adjuvant chemotherapy with gemcitabine. Interestingly, in this case, the Ki-67 proliferation index of sarcomatoid tumor cells was lower than that reported in other cases [26].

Of pivotal interest is the comparison made by the same authors between the aforementioned patient with the very long survival and the other two patients with SCP. The first case, treated with the same management, i.e., surgical resection followed by adjuvant chemotherapy, died after 18 months due to liver failure for multiple hepatic metastases. The other case was not amenable to radical resection and died 2 months after the clinical presentation. In all the three cases, a positive staining for phospho-Smad2/3, Snail, and fibronectin was observed at the IHC level in sarcomatoid tumor cells. However, the expression of senescence markers, such as γ-H2AX, p53, and p21, was identified only in the sarcomatoid compartment of the long-term survivor, thus suggesting a potential correlation with prognosis [27]. TGF-β is a master-inducer of EMT [28,29]. Intriguingly, phospho-Smad2/3 is a downstream effector of TGF-β pathway activation, which is known to upregulate p53 expression [30]. Collectively, the TGF-β/phospho-Smad/p53/p21 axis, associated with the concept of cellular senescence, seemed to be active only in the sarcomatoid cells of the long-term survivor. In agreement with this conclusion, the Ki-67 proliferation index of the long-term survivor was the lowest compared with the other two cases [27].

Sarcomatoid carcinoma could arise from various organs besides pancreas, such as the lungs, the kidney, and the liver. A set of FFPE tissues from 71 patients with sarcomatoid cancer with different origins was subjected to NGS analysis. Among them, there were three patients with SCP. In all these cases, *KRAS* and *TP53* mutations were detected. Moreover, additional molecular alterations were identified in other genes, including *CDKN2A*, *TSC2*, *ERBB4*, *ROS1*, *KIT*, and *PDGFRA* [31].

Regarding the therapeutic approach, it has to be acknowledged that most patients with SCP described in the literature have been treated with surgical resection and adjuvant chemotherapy, sometimes with significant benefit. However, new horizons in this field may be opened by immunotherapy, the new frontier of oncology treatments. It has drastically changed the therapeutic opportunities of numerous cancer types. However, immunotherapy in PC has demonstrated limited effectiveness so far, mostly due to the immunosuppressive tumor microenvironment (TME) of conventional PDAC [32,33,34,35]. Interestingly, a SCP patient progressed after several lines of chemotherapy and was eligible for an anti-PD-1 immune-checkpoint inhibitor (i.e., pembrolizumab) due to the presence of microsatellite instability (MSI). Thanks to this approach, the patient registered an extensive tumor regression and is still alive 3 years after the first administration of such treatment [36].

PD-L1 assessment, along with tumor mutational burden and microsatellite instability, is the only validated predictive biomarker for immunotherapy administration. Of note, a recent study tried to shed light into this complex scenario. By analyzing six cases of SCP, the authors calculated the IHC expression PD-L1 using a standardized score, i.e., the Combined Positive Score (CPS). It is a pure number derived indicating the number of PD-L1 staining cells (tumor cells, lymphocytes, and macrophages) divided by the total number of viable tumor cells, multiplied by 100. It is considered positive in the case of ≥1, but this threshold can vary based on tumor types and histological features. Interestingly, PD-L1 CPS ≥ 1 was frequent in SCPs (5/6); of note, three cases displayed very high values, with CPS > 50. Beside the PD-1/PD-L1 axis, the authors also investigated Notch signaling, since it has been demonstrated to influence TME composition and PD-L1 expression, with direct implications on immunotherapy effectiveness in different tumor types [37,38,39]. Of note, through IHC and immunofluorescence analyses, the authors showed that Notch1 and Notch 3, two of the most-important Notch signaling effectors, were strongly expressed in all cases. Moreover, the activation of Notch signaling in SCP was further demonstrated by the expression of Hes1, an important Notch-signaling target, and of Jag1, the most-important Notch ligand. Further immunofluorescence analysis showed that PD-L1 and Notch3 are co-localized in sarcomatoid cells [40]. All these findings identified a unique biological characterization of SCP, providing a rationale for future studies evaluating the potential crosstalk between the PD-1/PD-L1 axis and Notch pathways and prompting the development of novel immunotherapy-based strategies.

## 3. Conclusions

SCP is a rare subtype of PDAC and is generally considered a very-aggressive disease with a dismal prognosis. However, due to its rarity, its clinic-pathological parameters are still poorly characterized. Interestingly, in this review, we highlighted also the occurrence of cases that achieved more than 10 years of survival after surgical resection. Early diagnosis and surgical resection followed by PDAC-standardized adjuvant chemotherapy represents at this time the only concrete possibility for long-term survival. Further studies are needed to improve strategies for early SCP detection and to identify patients that can be treated with personalized treatments. SCP histology is well-characterized by the presence of undifferentiated cells with mesenchymal features. The SCP immunohistochemical profile is mainly characterized by the co-expression of epithelial markers such as cytokeratins and mesenchymal markers such as vimentin. Collectively, a standardization of the terminology and the criteria for an accurate SCP diagnosis should be established in order to correctly identify these rare cancers. Although the knowledge regarding the molecular mechanisms behind SCP oncogenesis and progression is still very limited, *KRAS* and *TP53* mutations seem to act as driver events in these tumor types. Collectively, large-scale genomic and transcriptomic studies based on larger SCP patients’ cohorts are warranted in order to describe in greater depth its molecular profile and to compare this rare cancer type with the most-important molecular PDAC subgroup, including those such as classical and quasi-mesenchymal subgroups. Of note, recent evidence that suggests a possible correlation between TGF-β1-mediated senescence and long-term survival should be interpreted as very promising findings. As here discussed, a non-negligible proportion of SCP patients could benefit in the near future from new immunotherapy-based strategies. Along this line, one of the most-promising novelties in SCP regarded the activation of the TME-modulating Notch signaling and its possible interaction with the PD-1/PD-L1 axis.

## Figures and Tables

**Table 1:** Summary of individual clinical cases of sarcomatoid carcinoma of the pancreas.

Case/Ref.	Age (Years)/Gender	Pancreas/Tumor Size (cm)	Surgery/Resection Margin Status	(Adjuvant) Therapy	Sarcomatoid Compartment	Follow Up/Survival
1 [17]	39/M	Head/7 × 10	-	-	Vimentin	1 month/died of disease
2 [18]	58/F	Tail/16 × 18	Tumor resection/R0	Chinese medicine and thymosin	Vimentin, CK7	5 months/developed metastases but still alive
3 [19]	48/M	Tail/10 × 8 × 5	Left pancreatectomy/R0	Gemcitabine	Vimentin	3 months/died of disease
4 [20]	64/F	Head/3.7 × 3.6	-	Palliative radiotherapy	Vimentin, CD56	3 months/died of disease
5 [21]	63/M	Head/2.5 × 2 × 1.8	Pancreatoduodenectomy/R0	Thymopeptides	Vimentin, CK7, CK19	18 months/died of disease
6 [22]	64/M	Head/2.4 × 2 × 1.9	Pancreatoduodenectomy with cholecystectomy/R0	Gemcitabine	Vimentin	19 months/alive
7 [23]	61/F	Tail/3.2 × 2.9	Pancreatectomy with splenectomy/R0	-	Vimentin, pan-CK	35 months/alive
8 [24]	67/F	N/A /4	Pancreatoduodenectomy/R2	-	N/A	2 months/died of disease
9 [24]	80/F	N/A /5	Pancreatoduodenectomy/R0	-	N/A	1 month/alive
10 [24]	63/F	N/A /5.7	Distal pancreatectomy/R1	-	N/A	1 month/died of disease
11 [24]	56/F	N/A /5	Total pancreatectomy/R0	Capecitabine	N/A	3 months/Alive
12 [24]	79/M	N/A /4	Pancreatoduodenectomy/R0	-	N/A	3 months/died of disease
13 [24]	54/M	N/A /3	Distal pancreatectomy/R0	Gemcitabine, capecitabine+ radiation	N/A	61 months/alive
14 [24]	65/M	N/A /15	Distal pancreatectomy/R0	-	N/A	3 months/died of disease
15 [24]	73/F	N/A /9	Pancreatoduodenectomy with total gastrectomy/R0	Radiation	N/A	188 months/alive
16 [25]	48/M	Tail/10 × 8 × 3.5	Tumor resection/R0	Gemcitabine Oxaliplatin Floxuridine	Vimentin AACT CK18 CK19 pan-CK	>36 months/alive
17 [26,27]	58/M	Body/ Diameter:5	Pancreatectomy with splenectomy/ N/A	Gemcitabine	Vimentin CK p-Smad2/3 Snail Fibronectin γ-H2AX p53p21	120 months/alive
18 [27]	68/M	N/A /Diameter:4	Distal pancreatectomy/ N/A	Chemotherapy	p-Smad2/3 Snail Fibronectin	18 months/died of disease
19 [27]	65/F	N/A /Huge tumor	-	Cisplatin	p-Smad2/3 Snail Fibronectin	2 months/died of disease

Abbreviations: Ref., reference; M, male; F, female; CK, cytokeratin; AACT, anti-alpha 1 antichymotrypsin; p, phospho.
